# Choice enhances touch pleasantness

**DOI:** 10.3758/s13414-024-02887-6

**Published:** 2024-06-10

**Authors:** Lenka Gorman, Wenhan Sun, Jyothisa Mathew, Zahra Rezazadeh, Justin Sulik, Merle Fairhurst, Ophelia Deroy

**Affiliations:** 1 Cognition, Values, Behaviour Lab, Ludwig Maximilian University of Munich, Munich, Germany; 2 Chair of Philosophy of Mind, Faculty of Philosophy, Philosophy of Science and Religious Studies, Ludwig Maximilian University of Munich, Munich, Germany; 3 Institute for Psychology, General and Experimental Psychology, Ludwig Maximilian University of Munich, Munich, Germany; 4 Faculty of Psychology, Ludwig Maximilian University of Munich, Munich, Germany; 5 Centre for Tactile Internet with Human-in-the-Loop (CeTI), 6G Life, Dresden University of Technology, Dresden, Germany; 6 Chair of Acoustics and Haptics, Faculty of Electrical and Computer Engineering, Dresden University of Technology, Dresden, Germany; 7 Munich Center for Neuroscience, Ludwig Maximilian University of Munich, Munich, Germany; 8 Graduate School of Systemic Neuroscience, Ludwig Maximilian University of Munich, Munich, Germany; 9 Institute of Philosophy, School of Advanced Study, University of London, London, UK

**Keywords:** Affective touch, Choice, Reward, Eye movement, Control

## Abstract

We value what we choose more than what is imposed upon us. Choice-induced preferences are extensively demonstrated using behavioural and neural methods, mainly involving rewarding objects such as money or material goods. However, the impact of choice on experiences, especially in the realm of affective touch, remains less explored. In this study, we specifically investigate whether choice can enhance the pleasure derived from affective touch, thereby increasing its intrinsic rewarding value. We conducted an experiment in which participants were being touched by an experimenter and asked to rate how pleasant their experience of touch was. They were given either a choice or no choice over certain touch stimulus variables which differed in their relevance: some were of low relevance (relating to the colour of the glove that the experimenter would use to touch them), while others were of high relevance (relating to the location on their arm where they would be stroked). Before and during touching, pupillometry was used to measure the level of arousal. We found that having a choice over aspects of tactile stimuli—especially those relevant to oneself—enhanced the pleasant perception of the touch. In addition, having a choice increases arousal in anticipation of touch. Regardless of how relevant it is to the actual tactile stimulus, allowing one to choose may positively enhance a person’s perception of the physical contact they receive.

**Supplementary Information:**

The online version contains supplementary material available at 10.3758/s13414-024-02887-6.

What we choose is more valuable to us than what is forced upon us (Egan et al., 2010; Sharot, De Martino et al., 2009a; Sharot et al., 2010). This conclusion is well documented by neuroscience research, which shows that active choice leads to increased brain activity in the striatum—an area associated with reward—and an increase in the release of dopamine, a neurotransmitter implicated in learning about the reward value of a stimulus (Ferreri et al., 2019; Fujiwara et al., 2013; Sharot, Shiner et al., 2009b). However, the impact of choice on intrinsically rewarding experiences, particularly in the domain of interpersonal touch, remains a less explored area. In recent years, while the literature on choice effects has grown rapidly, our focus here is to specifically investigate whether choice influences the perception of pleasant touch, with a particular emphasis on adult individuals.

Interpersonal touch is valuable to humans, both in development and throughout life. Touch has profound benefits for our physical and mental well-being (Drescher et al., 1980; Field, 2019; López-Solà et al., 2019; von Mohr et al., 2017), and its deprivation may result in depression, anxiety disorders, low self-esteem, and illness (Durkin et al., 2021; Smirni et al., 2019; von Mohr et al., 2021). More specifically, pleasant touch such as the act of hugging, cuddling, and holding hands can improve mood, reduce stress, and strengthen interpersonal relationships (Coan et al., 2006; Morrison, 2016). This interpersonal touch, intricately connected to emotional bonds, as highlighted by Suvilehto et al. (2015), forms a crucial component of the positive outcomes associated with touch. Moreover, many studies have identified pleasant touch as influencing a wide range of decision-making processes and preferences (Camps et al, 2013; Crusco & Wetzel, 1984; Hornik & Ellis, 1988). It is noteworthy, however, that the majority of these effects are predicated on the individual’s active choice of touch rather than its imposition.

If one follows the existing literature on pleasant touch, however, the importance of choice dissipates or is not directly manipulated. The first explanation is the importance of bottom-up factors arising from the discovery that pleasant touch strongly corresponds to the stimulation of C-tactile (CT) afferents. CT afferents are primarily identified in hairy skin (Vallbo et al., 1993) and are slow conducting mechanoreceptors, optimally responsive to gentle stroking velocity in the range of 1–10 cm/s (Ackerley et al., 2014; Löken et al., 2009). Further research has highlighted their significance in various aspects of social touch and its hedonic components (McCabe et al., 2008; Sailer et al., 2020). Numerous studies have examined the effect of stroking velocity on perceived pleasantness as modulated by CT afferents (Ackerley et al., 2014; Löken et al., 2009; Olausson et al., 2008). Additional studies have highlighted the importance of top-down factors and expectations yet have predominantly examined pleasant touch within experimental contexts where participants lacked both choice and control over the touch they received: Touch was controlled by the experimenters. Some studies, such as Perini et al. (2015), have shown that people will choose touch experiences that correspond to CT-optimal velocities (1–10 cm/s) over the very slow or very fast speeds less likely to activate CTs (0.3 or 30 cm/s). However, such findings are more related to showing that people want strokes that tap into the CT system, rather than examining whether choice impacts how much people find touch pleasant. This leaves us with unanswered questions about whether providing participants with a choice amplifies the perceived pleasantness of the touch.

## Why choice matters

Since choice is such an expansive and multifaceted concept, it becomes vital to elucidate its intricate and multifaceted aspects in our project context. In this experimental situation as well as in clinical ones, people need to give consent to being touched by the scientist or doctor in charge. Once this overall consent is provided, people are offered a choice of the location or the way they will be touched: This is the choice in which we are mostly interested. Although choice is not the same as control, choice is often causally linked to control in the sense that it provides the ability to bring about positive outcomes or to prevent negative effects through one's own actions (Averill, 1973; Inesi et al., 2011). Perceived control is in turn important for personal satisfaction and beneficial for human flourishing (Leotti et al., 2010).

Social psychologists and other disciplines interested in the “illusion of control” in decision-making (for review, see Wang et al., 2021) have documented the occurrence of choice-induced preferences, where the act of choosing can change our disposition toward the chosen options. In the “free-choice paradigm,” Brehm (1956) showed that people reported more liking for the option they had chosen than they did before choosing it—an effect which can be traced in the brain (Izuma et al., 2010) and can last for several years (Sharot et al., 2012).

One plausible explanation is that the resulting perception of control is intrinsically rewarding (Leotti & Delgado, 2011; Wang & Delgado, 2019). In that case, choice would boost experienced liking or pleasantness because of the intrinsic rewards associated with increased personal control. However, an alternative explanation is that choice reduces uncertainty and increases predictability, which are also positive experiences (Lee & Daunizeau, 2020). If so, the choice could still be beneficial, but without this necessarily explaining any direct boost to the reward value of the perceptual experience.

In light of these disagreements, our aim is to examine whether choice impacts the perceived pleasantness of touch and does so because of its boost of the intrinsic reward of touch, which, in the context of our discussion, relates to the anticipation of touch experiences. While we recognize the connection between choice and a sense of control, our focus is on understanding how choice specifically impacts the perceived pleasantness of these experiences. We do not directly measure the sense of control itself, but rather we explore whether choice extends its influence to rewarding experiences like affective touch.

It is worth noting that while prior research has primarily concentrated on the effects of choice on objects, such as food, money, or material goods, which can be counted as extrinsic rewards, our shift to touch shifts the attention to how choice influences the perception of intrinsically rewarding experiences, such as affective touch. Intrinsic rewards are heterogeneous and defined as those that are enjoyed with no immediate survival benefit (Blain & Sharot, 2021), in contrast to primary rewards (e.g., water, food) and secondary rewards, which are associated with them (e.g., money). This does not rule out that, evolutionarily or in development, something became intrinsically rewarding because of its association with a primary reward. However, it can ultimately be enjoyed without having any survival benefit. Hence, affective touch is here a plausible candidate for an intrinsically rewarding experience.

## Study outline

We examined how the perception of touch pleasantness is modulated by choice, measuring both subjective and physiological responses. On the first hand, we tested whether having a choice would alter participants’ reported pleasantness of touch. To make sure that we isolated the effect of choice, we compared two scenarios: one in which the choice related to a feature not relevant to touch (the colour of the glove touching the participant, where different-coloured gloves would feel the same) and one where the choice was of high relevance (the place where the participant's arm would be touched, which could have some personal relevance). To determine whether the effect was specific to pleasant touch, or whether having a choice would be sufficient to increase the pleasantness of any touch, we compared two speeds of touch which differentially activate CT afferents as emphasized in studies by Ackerley et al. (2014) and Löken et al. (2009): one CT-optimal (3 cm/s) and the other CT-suboptimal (30cm/s).

To ensure that our results are not purely a matter of subjective reporting, we also explored the effects of the above experimental manipulations on lower-level physiological measures. It is well-established that pupil dynamics are physiological markers of arousal (Chang et al., 2016; Sirois & Brisson, 2014; Urai et al., 2017), a state of physiological activation through external and internal stimulation (Strauch et al., 2022). In this study, we explored changes in pupil size dynamics as an indicator of arousal to investigate the impact of choice on both tactile perception and anticipation. While arousal has a greater effect on pupil dilation than valence does (Mathôt et al., 2018), a few studies have also examined the relationship between perceived touch pleasantness and arousal (Gusso et al., 2021), so we also report the findings that speak to this relationship here. In doing so, our study provides a more comprehensive understanding of the underlying mechanisms involved in the impact of choice on touch perception. It is our belief that this combined approach can provide valuable insight into how touch perception and physiological responses are interconnected.

We hypothesized that providing individuals with a choice in touch experiences will lead to increased perceived pleasantness compared to situations where touch is not chosen, independently of whether the choice is highly relevant or not. In addition, we hypothesized that choice will also influence the physiological responses associated with arousal. Specifically, we were interested in seeing whether participants, when given a choice regarding touch, would exhibit different pupil size dynamics during the anticipatory and/or stimulation phases compared to when they received the same stimulation with no choice. The temporal aspect plays a pivotal role in elucidating how choice influences the perceived pleasantness of touch. This investigation probes whether the observed impact of choice on perception, as indicated by both the main effect and interaction with relevance in our models, arises from differences in anticipating upcoming sensations or is solely attributed to heightened arousal during the experience. Importantly, our findings highlight the presence of a nuanced relationship between choice, relevance, and pupil sizes, revealing that evidence for both aspects coexists without being mutually exclusive. .

## Methods

All the experimental procedures were approved by the University of the Bundeswehr Munich Research Ethics Board. Detailed study information was given to all participants and written consent was obtained. The entire experiment was conducted in German, including all the questionnaires. Participants were compensated for their time. Data from electrocardiogram and electrodermal activity were collected as well, though they are intended for a different research question and are not presented here.

### Participants

Twenty-five healthy volunteers were recruited for this study through the Bundeswehr University Munich email distribution list. Due to the incomplete recording of pupil-size data, two participants were excluded from the final analysis. Ultimately, the results of data analysis from 23 participants (5 women and 18 men) are reported here. Participants were all native German speakers, right-handed, did not wear glasses, between the age of 18 and 35 years, and had a university degree or were pursuing one. The sample size was constrained by the challenges of recruiting for a study that required close contact during the COVID-19 pandemic.

### Questionnaires

As part of study enrolment, participants completed a demographic questionnaire, which included information about gender and education. To control for possible differences in the initial attitudes toward physical touch, participants filled out the Social Touch Questionnaire (STQ; Wilhelm et al., 2001), and to understand their perception of touch behaviour, we included The Longing for Interpersonal Touch Picture Questionnaire (LITPQ; Beßler et al., 2020). These questionnaires have a well-established history of assessing psychological and emotional factors in affective touch perception, confirming their reliability and relevance. For further details about these scales, see https://osf.io/z7a9h.

Because the experiment was conducted at a time when the pandemic was ongoing, and because people may have been afraid of touch, on the day of the experiment, participants also completed the six-item short form of the Spielberger State-Trait Anxiety Inventory (STAI-6; Marteau & Bekker, 1992), both before and after the experiment. This was included as a control variable as anxiety about the experiment might impact reported perceptions of touch.

### Participant setup

**Fig. 1:**
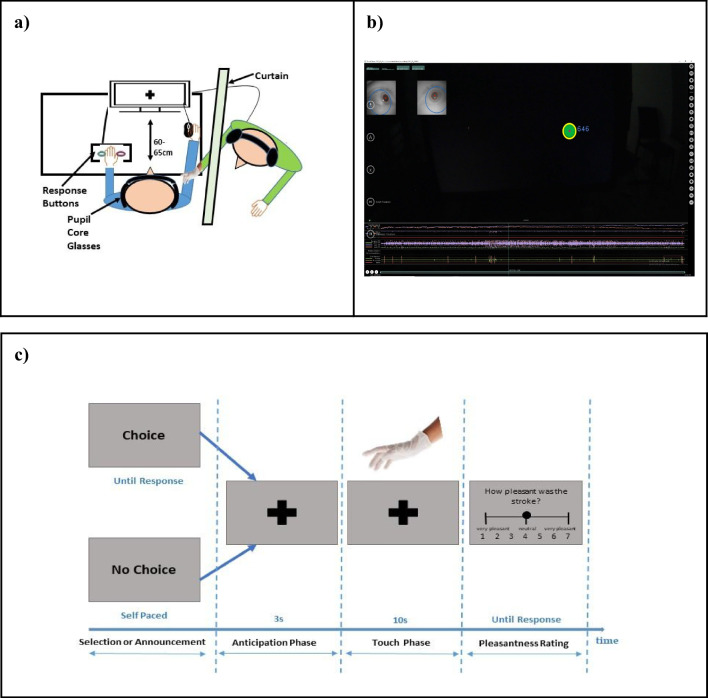
The experiment settings and its time course. *Note*. **a)** shows the experiment settings applied to the PupilCore; **b)** demonstrates the pupil size recordings; **c)** illustrates the time course of the events of the experiment: Every frame represents a screen presented to a participant. (Colour figure online)

Participants entered the darkened room, upon which their written consent was obtained and the areas of their arms where they would be touched were marked. They then sat comfortably facing the computer screen (Fig.1a), at 60–65 cm from the monitor, while resting their right hand on the table and having their left hand in easy reach of the response buttons placed on the table. The Pupil Core, our physiological recording device, was introduced and calibrated in the darkened room. Importantly, the darkened environment and the participants’ distance to the monitor stayed stable throughout the recording, to ensure factoring out pupillary light responses and pupillary near responses, respectively. The experimenter sat behind a curtain, opposite and to the right of the participant so that only their stroking arm/hand was visible to the participant through the curtain.

### Experimental design

Prior to the main experiment, a pretesting pilot phase was conducted to ensure uniform touch perception between the left and right hand. This precautionary measure aimed to verify that potential variations in touch sensations experienced by participants were minimized, supporting the internal validity of our study.

The experiment consisted of two practice trials followed by 96 experimental trials. We used a 2 × 2 × 2 factorial design for our experiment. In each trial of the experiment participants either made a choice or a choice was made for them (Factor 1: choice vs. no choice). The choice was either relevant to the participant’s experience of being touched by the experimenter or not (Factor 2: high relevance vs. low relevance). Specifically, participants chose whether they wanted to be touched on their upper or lower arm in the high-relevance condition. In contrast, in the low-relevance condition, they chose the colour of the glove worn by the experimenter (blue or white, but of the same texture and thus not affecting tactile experience). Finally, the touch was either delivered at a CT-optimal speed where the arm was stroked by the experimenter’s hand moving at 3 cm/s or at a CT-non-optimal speed of 30 cm/s (Factor 3: optimal vs. suboptimal speed).

Thus, in each trial (Fig.1c), participants selected either the site of contact or the colour of the stroking glove, or else they were just informed about these parameters, depending on the random condition selected. For instance, in a high-relevance trial, participants either chose or were informed about the site of contact (upper or lower arm), while the glove colour was predetermined (white). Conversely, in a low-relevance trial, participants selected or were informed about the colour of the glove worn by the experimenter, while the site of contact was predetermined (lower arm). This approach allowed us to systematically investigate the impact of choice on specific aspects of the touch experience while keeping other factors constant or randomly assigned. Participants were not provided with simultaneous information about all aspects in a single choice.

Once the randomly assigned condition was established, it was followed by 13 s of a central fixation cross, consisting of 3 s of stroke anticipation and 10 s of stroking. Subsequently, participants had 4 seconds to respond and rate “How pleasant was the stroke” on a 7-point Likert-like scale, ranging from 1 (*very unpleasant*) to 7 (*very pleasant*), with 4 representing *neutral*. Once the rating was submitted, the next trial promptly commenced.

The experiment consisted of four randomized blocks based on our 2 × 2 × 2 factorial design, with 24 trials within each block. Participants took a 1-minute break between blocks.

### Stimuli and touch administration

We marked each stroking area on the participant’s right anterolateral upper arm and forearm with a length of 10 cm. Depending on the experimental condition (Fig.1c), each participant was stroked by an examiner’s latex-gloved hand with either of the two different velocities (3 or 30 cm/s) in a proximal-to-distal direction for a period of 10 s, employing a gentle stroke technique that could be described as akin to the sensation of gentle four-fingers stroking.

During the experiment, two male experimenters alternated between the roles of toucher and equipment manager. Though participants had brief interactions with the equipment manager before the experiment, during the actual stroking phase, the toucher remained hidden behind a screen, out of view of the participant. Participants could only see the toucher’s gloved hand used for the stroking procedure. The touchers underwent thorough training to proficiently deliver touch with both differently coloured gloves. We also performed pretesting to confirm uniform touch perception regardless of hand used by the experimenter, reducing potential variations in participants’ touch experiences.

In our experiment, the experimenter wore a white latex glove on the right hand and a blue latex glove on the left hand. The choice of glove colours was deliberate and served as part of our experimental design. The experimenter was seated to ensure comfortable access to the participant’s arm, facilitating consistent and controlled strokes from both arms.

Cues for the toucher’s actions were conveyed through headphones, using a prerecorded voice and metronome sound, indicating the current condition and stroke speed within the randomly assigned block. The stroke speed was not disclosed to the participant before.

This setup was designed to minimize any potential differences in the stroking experience between the two arms, ensuring that the touch sensations were consistent and as similar as possible.

### Recording and apparatus

The physiological responses (pupil size data) were recorded in a darkened room as the task procedure was controlled through a custom Python script running in PsychoPy (Peirce et al., 2019). A Pupil Core wearable eye tracker (Kassner et al., 2014; Pupil Labs, 2021) was used to measure binocular pupil size in millimetres. This apparatus comprises two eye cameras (200 Hz each) and a scene camera (60 Hz). The Pupil Capture Software (Pupil Labs, 2021), as shown in Fig. 1b, provided a real-time pupil-size tracking algorithm to detect pupil shape and position. A fixation cross was displayed in the middle of the screen during the anticipation and touch period (Fig. 1c), to ensure good pupil data recording.

### Pupil-size data preprocessing

Pupil Player software (Pupil Labs, 2021) was used to visualize and export the data. The preprocessing steps were performed in Python and consisted of the rejection of invalid values (out of the 1 to 9 millimetres biologically possible range), blinks, values recorded with low device reliability (as reported by the device software) and values recorded with excessive dilation speed (also biologically impossible), after which interpolation was performed. The clean time series data was then segmented based on experimental triggers, *z*-scored, resampled and baseline-corrected (Van Slooten et al., 2018). Each trial consisted of a 3-s anticipation phase, followed by 10 s of stroking and 4 s pleasantness response time (Fig. 1c). The data processing script can be found at https://osf.io/jgknx.

### Analysis strategy

Linear mixed-effects regressions (LMERs, R Package lme4; Bates et al., 2015) were used to determine whether and how subjective perception of touch pleasantness was affected by the participant being given a choice (choice/no choice), the relevance of choice outcome (high/low), and the speed of the stroke (optimal/suboptimal). The model included two- and three-way interactions between these factors, as well as a random intercept for the participant and by-participant random slopes for each of the main predictors (choice, relevance, and speed). The model thus has a maximal random effects structure (Barr et al., 2013). Further, as the overall effects of choice, relevance or speed—or the effects of one of these at average levels of the others—are not readily interpretable with only the raw regression coefficients (especially given the inclusion of a three-way interaction), we use the R package marginaleffects (Arel-Bundock, 2023) to estimate these effects. In supplemental analyses, we also report equivalent results with cumulative regression models (R package ordinal; Christensen, 2022). 

To model pupil size during the anticipation and touch phases, given nonlinearity and autocorrelation in pupil size over time, we used generalized additive mixed-effects models (GAMMs, R package mgcv; Wood, 2011) with similarly maximal random effects structures. One difference between the anticipation and touch phases was that, as there was no tactile stimulus during the anticipation phase, touch speed was not included as a predictor of pupil size during this phase. For an analysis of pupil size across anticipation and touch phases, raw pupil sizes were normalized via *z*-scoring. Then, to isolate effects specific to each trial stage, the *z*-scored pupil sizes were baseline-corrected by subtracting the mean size of the final 1 s of the previous phase (for the anticipation phase, the final 1 s of the selection/announcement phase in Fig. 1; for the touch phase, the final 1s of the anticipation phase).

Finally, we used LMERs to investigate any relation between pupil dynamics and perceived pleasantness. For all LMERs, *p* values were provided by the package lmerTest (Kuznetsova et al., 2017). 

Across all models, STQ, LITPQ and STAI-6 survey scores were included as covariates. In Appendix 1, we briefly describe the questionnaires we used and the appropriate interpretation of their scores.

## Results

### Behavioural outcomes

#### Does having a choice, varying degrees of relevance, and speed of tactile stimuli affect the perception of pleasantness?

**Fig. 2:**
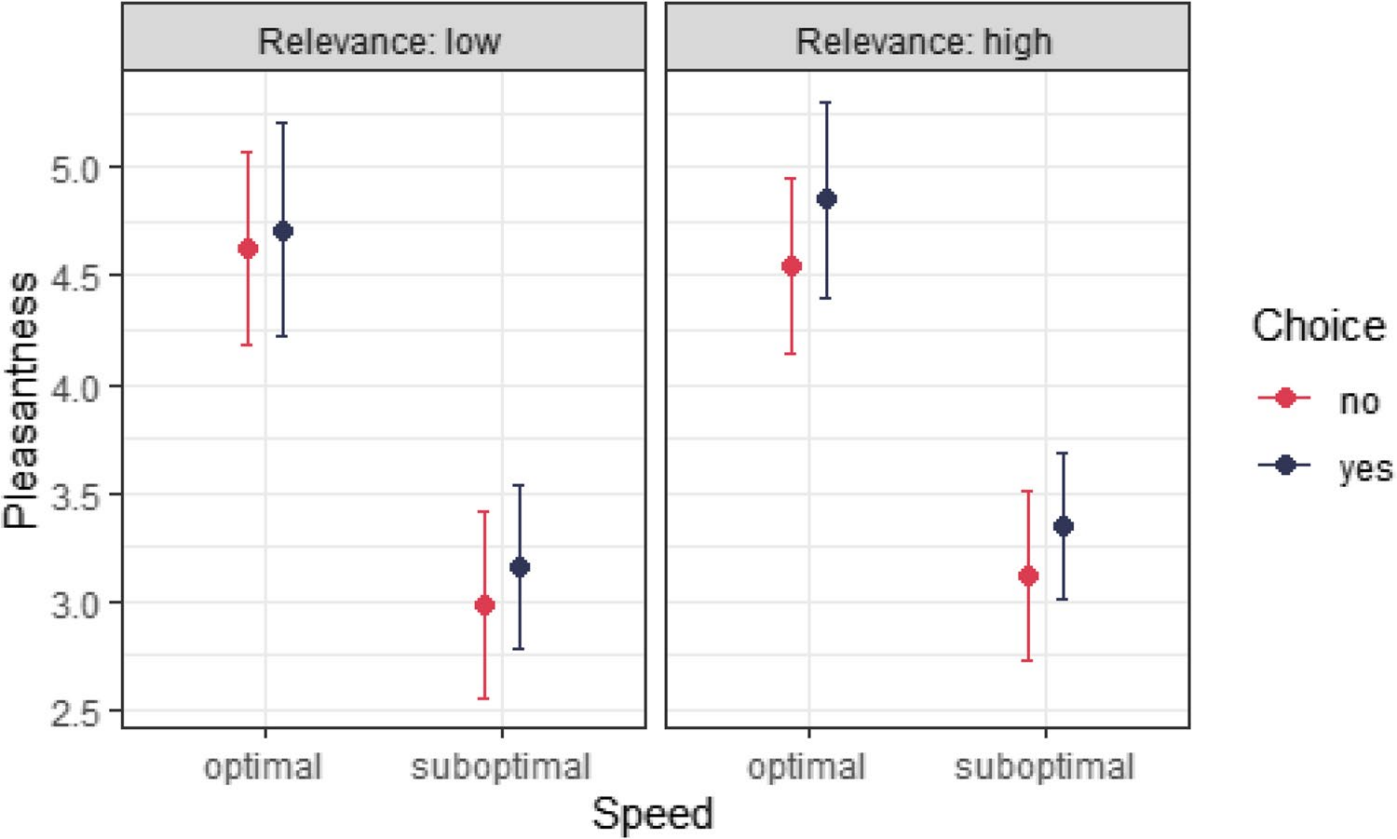
The effect of choice, relevance, and speed on pleasantness. *Note.* Model-estimated means and 95% confidence intervals (CIs) for perceived pleasantness as a function of speed, choice and stimulus relevance. (Colour figure online)

We regressed pleasantness on choice (choice/no choice), speed (optimal/suboptimal to CT), and relevance (high relevance, arm site/low relevance, glove colour), including interactions between all three factors. For details on control variables and random effects, see the Analysis Strategy section, above. The model coefficients are shown in Table S1 (note that baseline/reference levels were speed = optimal, relevance = low, choice = no). Figure 2 plots the model-estimated mean pleasantness for each combination of conditions.

Unsurprisingly, given Fig. 2, there is an overall effect of speed (averaging over relevance and choice): CT-suboptimal stimuli were rated as less pleasant than CT-optimal ones (*b* = −1.53 [−2.1, −0.97], *SE* = 0.287, *z* = −5.34, *p* < .001). This phenomenon has been documented in a number of studies that demonstrate the role of CT fibre stimulation in causing an affective touch response (Löken et al., 2009; Olausson et al., 2008; Pawling et al., 2017). There is also an overall effect of choice (averaging over relevance and speed): participants typically rated touch as more pleasant when they were given a choice (*b* = 0.194, [0.052, 0.335], *SE* = 0.072, *z* = 2.69, *p* = .007). There was no overall effect of relevance (averaging over speed and choice, *b* = 0.094 [−0.030, 0.219], *SE* = 0.064, *z* = 1.48, *p* = .138).

There was a significant interaction between choice and relevance (averaging over speed) such that merely having a low-relevance choice did not significantly increase perceived pleasantness (*b* = 0.127 [−0.026, 0.279], *SE* = 0.078, *z* = 1.63, *p* = .103), but having a high-relevance choice did (*b* = 0.261 [0.108, 0.413], *SE* = 0.078, *z* = 3.35, *p* < .001). Indeed, the effect of choice on pleasantness was significantly greater in high-relevance conditions than in low-relevance ones (*b* = 0.134 [0.019, 0.249], *SE* = 0.059, *z* = 2.29, *p* = .022). While choice had a positive effect on pleasantness both at CT-optimal and CT-suboptimal speeds, this effect of choice was not significantly different at different speeds (averaging over relevance, *b* = 0 [−0.115 0.115], *SE* = 0.059, *z* = 0, *p* = 1).

Finally, relevance’s boost to the effect of choice—the difference in rated pleasantness between being given a high-relevance choice and a low-relevance one relative to no choice—was significant at CT-optimal speeds (*b* = 0.221 [0.059, 0.383], *SE* = 0.083, *z* = 2.67, *p* = .008) but not at CT-suboptimal ones (*b* = 0.047 [−0.115, 0.209], *SE* = 0.083, *z* = 0.569, *p* = .569). However, in the absence of a significant three-way interaction (*b =* 0.174 [0.006, 0.343], *SE* = 0.117, *z =* 1.486, *p* = .137), we stop short of concluding that the former effect is significantly larger than the latter.

Overall, then, CT-suboptimal speeds were detrimental to perceived pleasantness of touch across conditions, while choice mattered when the choice was relevant to touch (here, where on the arm one was touched), and not when it was irrelevant (here, gloves of different colours but the same texture).

For the sake of robustness, as perceived pleasantness was rated on a Likert scale, we additionally ran alternative regression models apt for ordinal variables: cumulative mixed-effects models (using R package ordinal; Christensen, 2022). The model coefficients are reported in Table S2 and further details are provided at https://osf.io/jgknx/. These show the same patterns of significance as the more parsimonious and straightforwardly interpretable Gaussian regression reported above. 

We also report additional models that include participant gender as a covariate (both with and without interactions between gender and the experimental conditions) in Table S3 and at https://osf.io/jgknx/. However, the inclusion of gender as a predictor worsens model fit (ΔBIC = 7.9 for gender as a main effect, and ΔBIC = 50.9 for gender interacting with experimental conditions, relative to the main model reported above), and it does not alter our main conclusions.

### Pupillometry outcomes

#### Do choice and relevance contribute to anticipatory arousal before touch?

To model pupil size over the time course of the anticipation phase of each trial, we regressed pupil size (both *z*-scored pupil size—to provide continuity over trial phases—and *z*-scored baseline-corrected pupil size, for effects specific to each trial phase) on choice and relevance. The GAMMs included a smooth term for time (with the difference smooths for each level of choice and of relevance), random intercepts for trial and participant, by-participant random slopes for choice and relevance, and a by-participant random smooth (see https://osf.io/jgknx for exact model specifications, including smooth terms).

**Fig. 3:**
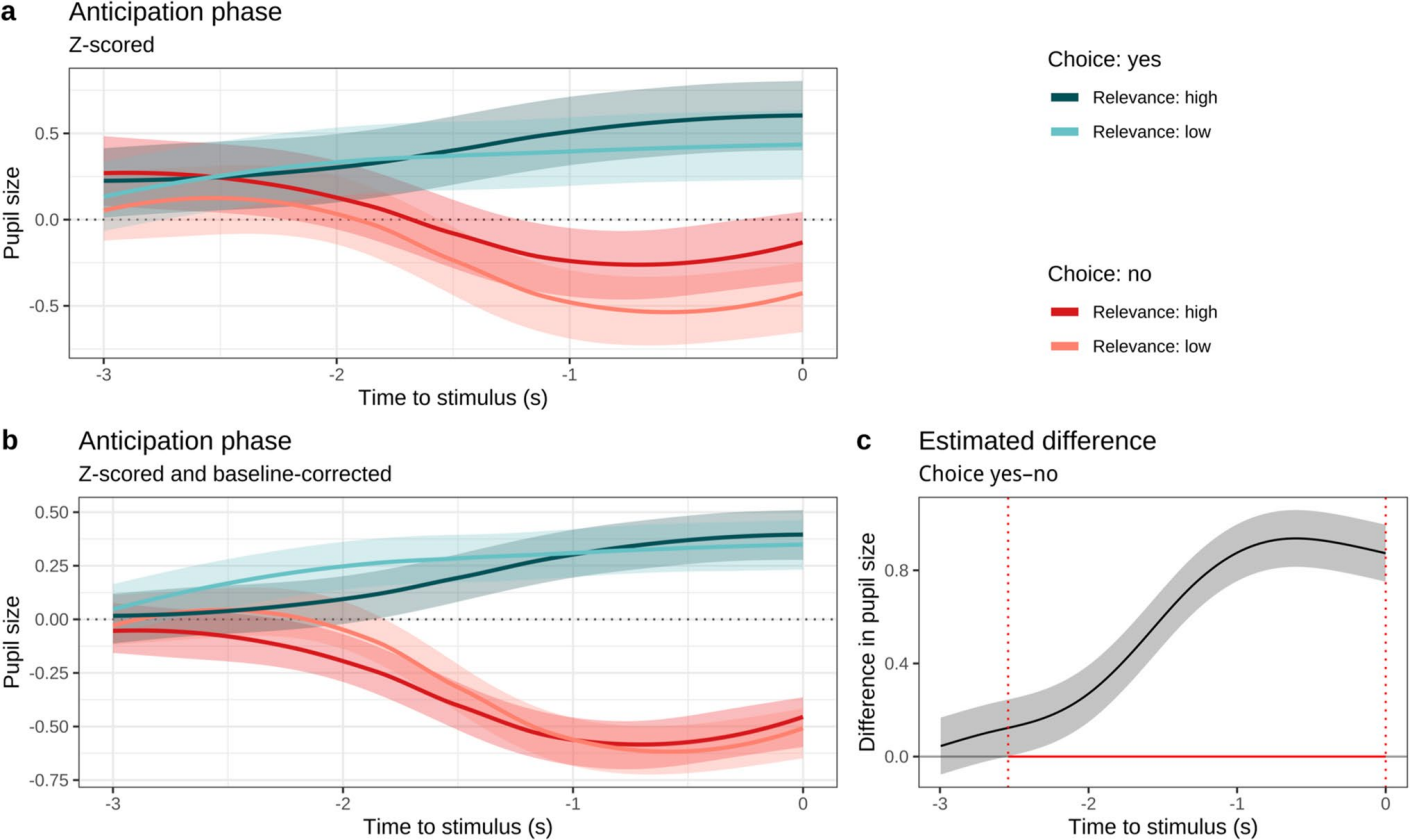
Model estimated pupil size in the anticipation phase as a function of choice and relevance. *Note*. **a** GAMM estimates of *z*-scored pupil size with 95% CIs; **b** GAMM estimates of *z*-scored and baseline-corrected pupil size (subtracting the average pupil size of the final 1 s of the preceding selection/announcement phase) with 95% CIs; **c** GAMM estimate of the difference between choice and no-choice conditions from **(b)**, illustrating how the difference is significant and positive, in that the 95% CIs lie above 0, from time = −2.541 until time = 0. The red line segment at *y* = 0 and dotted vertical lines mark the time period where the difference is significant. For model coefficients corresponding to subfigure **(a)**, here, see Table S4; for coefficients corresponding to subfigure **(b)**, see Table S5. For further details, see https://osf.io/jgknx/. (Colour figure online)

Figure 3a–b shows the mean model-estimated pupil sizes for each condition over the 3 s of the anticipation phase, illustrating a clear effect of choice in the later time periods of the trial.

For the *z*-scored outcome (Fig. 3a), relative to reference levels (no choice, low relevance), there was a main effect of choice (*b* = 0.558, *SE* = 0.088, *t* = 6.309, *p* < .001) and a significant interaction between choice and relevance (*b* = −0.125, *SE* = 0.003, *t* = −43.632, *p* < .001, so the difference between high-relevance and low-relevance trials was smaller in choice trials than in no-choice trials). For the *z*-scored and baseline corrected outcome (Fig. 3b, with the same reference levels) there is a similar pattern of results: a main effect of choice (*b* = 0.553, *SE* = 0.062, *t* = 8.911, *p* < .001) and a small though significant interaction between choice and relevance (*b* = −0.006, *SE* = 0.002, *t* = −2.491, *p* = .013). For both models, no other parametric coefficients were significant (for parametric coefficients, see Tables S4 and S5 or; for more details including smooth terms, see https://osf.io/jgknx/).

Figure 3c plots the difference between choice and no-choice trials over time (for the baseline-corrected model in Fig. 3b) and shows that pupil sizes were significantly larger in the choice condition from time = −2.541 s until the end of the anticipation phase (time = 0).

#### Do choice, relevance, and speed contribute to arousal during touch?

**Fig. 4:**
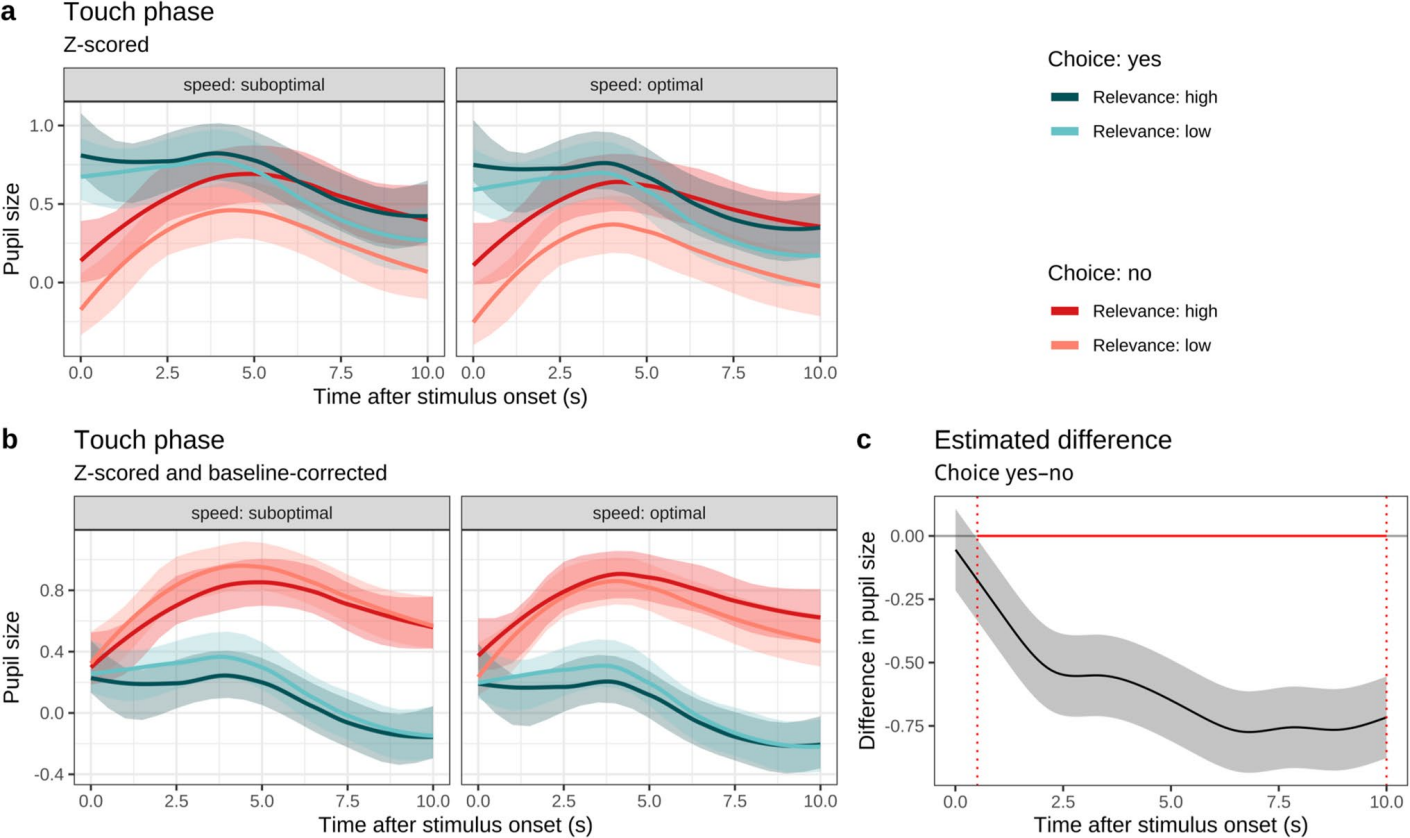
Model estimated pupil size in the touch phase as a function of choice, relevance, and speed. *Note*. **a** GAMM estimates of *z*-scored pupil size with 95% CIs, continuing the choice/no-choice difference seen at the end of the anticipation phase in Fig. 3; **b** GAMM estimates of *z*-scored and baseline-corrected pupil size (subtracting the average pupil size of the final 1 s of the preceding anticipation phase) with 95% CIs; **c** GAMM estimate of the difference between choice and no-choice conditions in **(b)**, illustrating how from time = 0.509 s until time = 10 s the difference is significant and negative, in that the 95% CIs lie below 0. The red line segment at *y* = 0 and dotted vertical lines mark the time period where the difference is significant. For model coefficients corresponding to subfigure **(a)**, here, see Table S6; for model coefficients corresponding to subfigure **(b)**, see Table S7. For further details, see https://osf.io/jgknx/. (Colour figure online)

Figure 4a–b shows the mean model-estimated pupil sizes for each condition over the 10 s of the touch phase. Figure 4a shows *z*-scored pupil sizes for continuity: As the pupil sizes in choice conditions were larger at the end of the anticipation phase in Fig. 3a, they started out larger here in the touch phase. Figure 4b shows baseline-corrected pupil sizes, to identify changes specific to this trial phase. The models were the same as for the anticipation phase, except that they also included touch speed (along with interactions with choice and relevance).

For the *z*-scored outcome in Fig. 4a (relative to the reference levels: no choice, low relevance, suboptimal speed) there was a significant main effect of choice (*b* = 0.315, *SE* = 0.079, *t* = 4.151, *p* < .001), a significant main effect of relevance (*b* = 0.259, *SE* = 0.094, *t* = 2.755, *p* = .006), and a significant main effect of speed (*b* = −0.103, *SE* = 0.039, *t* = −2.638, *p* = .008). There was an interaction between choice and relevance (*b* = −0.175, *SE* = 0.002, *t* = −72.649, *p* < .001, so the difference between low and high relevance was smaller in choice trials than in no-choice trials); an interaction between relevance and speed (*b* = 0.051, *SE* = 0.002, *t* = 21.153, *p* < .001, so relevance had a more positive effect when speed was optimal in the no-choice condition) and a three-way interaction between choice, relevance and speed (*b* = −0.028, *SE* = 0.003, *t* = −8.085, *p* < .001, so the aforementioned relevance: speed effect was weaker in choice trials). There were no other significant parametric effects (for parametric coefficients, see Table S6 or, for more details, https://osf.io/jgknx/).

For the z-scored and baseline-corrected outcome in Fig. 4b (with the same reference levels as above), there was a main effect of choice (*b* = −0.601, *SE* = 0.082, *t* = −7.322, *p* < .001, so in terms of effects attributable to the onset of touch stimulus, pupil sizes were higher in the no-choice conditions than in the choice conditions, given how baseline-correction counteracts the different endpoints in Fig. 3a–b); and a main effect of speed (*b* = −0.112, *SE* = 0.039, *t* = −2.853, *p* = .004, so optimal speed predicted smaller pupil sizes in no-choice, low-relevance trials). There were significant interactions between choice and speed (*b* = 0.030, *SE* = 0.002, *t* = 13.324, *p* < .001, so optimal speed had a more positive effect in choice trials than in no-choice trials when relevance was low); between relevance and speed (*b* = 0.165, *SE* = 0.002, *t* = 72.495, *p* < .001, so optimal speed had a more positive effect in high-relevance trials than in low-relevance trials when there was no choice), and a three-way interaction between relevance, speed and choice (*b* = −0.114, *SE* = 0.003, *t* = −44.634, *p* < .001, so the aforementioned speed–relevance interaction was weaker in choice trials than in no-choice trials). There were no other significant parametric effects (for parametric coefficients, see Table S7 or, for more details, https://osf.io/jgknx/).

As an alternative to GAMMS as models of nonlinear change over time, we also analyzed single time points per trial. Specifically, we performed peak detection (script provided at https://osf.io/jgknx) to identify the first peak in each trial after 220 ms, as this is the earliest plausible time for a pupil reaction after stimulus onset (Mathôt et al., 2018). This allows for LMER models that are simpler than the aforementioned GAMMs, but it assumes that the interesting effects are constrained to a particular time period. We regressed peak height for each trial on choice, relevance and speed (again, with interaction terms) with LMERs.

For the *z*-scored pupil values (relative to the same reference levels: no choice, low relevance, suboptimal speed), there was a main effect of choice (*b* = 0.405, *SE* = 0.103, *t* = 3.917, *p* < .001). All other effects were non-significant (see Table S8). For the *z*-scored baseline-corrected values, there was once more a main effect of choice, but this time negative (*b* = −0.462, *SE* = 0.084, *t* = −5.484, *p* < .001). There was also a significant interaction between choice and speed (*b* = 0.174, *SE* = 0.081, *t* = 2.151, *p* = .032) such that the difference between suboptimal speeds and optimal speeds was larger in the choice condition than it was in the no-choice condition. All other effects were nonsignificant (see Table S9).

Overall, this pattern of results points to there being an anticipatory effect of choice, but as Fig. 4a versus Fig. 4b illustrate, the main difference localizable to the touch phase itself is that the no-choice pupil sizes increase to eventually catch up with pupil sizes in the choice conditions.

#### How do anticipatory and touch arousal relate to perceived pleasantness in our conditions?

Additionally, we examined the relationship between self-reported pleasantness ratings and pupil size in the anticipatory and touch phases (taking pupil size to be the first peak poststimulus onset in the touch phase). Our findings showed that pleasantness and pupil size were not significantly related (*b* = −0.073, SE = 0.049, *t* = −1.495, *p* = .149).

## Discussion

Human behaviour is significantly influenced by choice, and our investigation into whether choice impacts perceived pleasantness of touch, an intrinsically rewarding stimulus, aligns with this understanding.

Our findings reveal that people perceive touch as more pleasant when they have a choice over certain aspects, with a notable impact on highly relevant factors, like choosing the location of the touch. This aligns with insights from Suvilehto et al. (2015), briefly highlighting the intricate relationship between social touch and emotional bonds among human adults. They showed that the total bodily area allowed for touching is linearly dependent on the emotional bond with the toucher across diverse cultures.

The positive effects of choice on pleasantness extend beyond individual preferences and operate within the broader context of social bonding. Our research indicates that these effects are more pronounced for highly relevant aspects such as choosing where one will be touched, but they can also be observed for less relevant choices such as selecting the colour of the glove used by the experimenter (though to a smaller extent). Our study reveals that not allowing choice results in significantly lower pleasantness scores, emphasizing the need to revisit experiments assessing touch pleasantness in paradigms without choice, as providing participants with a choice may yield even higher pleasantness than currently documented. The intricate connections between touch experiences and psychological and social factors, as highlighted by Suvilehto et al. (2015) and supported by our study, underscore the necessity for further exploration in this domain.

When we looked at the effect of choice on pupil size, we found that choice heightened peoples’ arousal in anticipation of touch. We observed a positive anticipation for more controlled, predictable stimuli, whether in their location or the less relevant aspect of colour. A lack of choice was associated with lower overall arousal, which may reflect a lower engagement with the forthcoming touch (Laeng et al., 2012). Higher levels of arousal were recorded during stroking after exercising choice, potentially suggesting that the participant was more aware of the event at hand when he/she participated in its occurrence. However, this may be a continuation of the aforementioned anticipation effect, as baseline-correction (to isolate the effects of touch during the touch phase) showed increased arousal in the no-choice condition. In brief, touch stimulus was associated with arousal (as indexed by pupil size), but being given a choice over the stimulus yielded anticipatory arousal, in which case the onset of the touch stimulus made little further difference.

Considering that pupil size is largely determined by arousal rather than valence (Mathôt et al., 2018), a lack of correlation between pupil size and reported pleasantness is not surprising. In addition, this finding is also in line with Strauch et al. (2022), suggesting that pupil size is determined more by how stimuli feel versus how they affect participants. Although only a few studies have been conducted in this domain thus far, the results further support Strauch et al. (2022). Building upon this emerging understanding, the study by van Hooijdonk et al. (2019) underscores the connection between touch intensity and pupil size, with their investigation revealing a heightened pupil response to CT-suboptimal brushing compared to CT-optimal brushing. Additionally, Bertheaux et al. (2020) demonstrated that perceived emotional intensity modulates pupil size based on texture exploration. It is worth noting that while Ellingsen et al. (2014) found a correlation between pleasantness of touch and increased pupil size, this relationship was observed in conjunction with the viewing of happy faces.

In light of this discussion, the study by van Hooijdonk et al. (2019) merits further exploration, particularly regarding the arousing nature of fast stroking touch, which may be attributed to other sensory afferents such as activation of A-beta mechanoreceptors (Löken et al., 2009). This mechanistic perspective could be examined more comprehensively in future research to deepen our understanding of touch-induced arousal.

Overall, the observed larger pupil size in the choice condition contributes to our broader understanding of how choice influences the perception of intrinsic rewards, like affective touch, emphasizing the complex interplay between sensory experiences and psychological factors.

Importantly, our study suggests that the effect of choice on liking previously observed for extrinsic rewards also extends to intrinsically rewarding experiences, such as affective touch. As such, our findings point to possible ways to improve touch perception. Touch influences our everyday life choices, such as our behaviour toward other people (Guéguen, 2004), our risk preferences (Levav & Argo, 2010), and our economic decisions (Crusco & Wetzel, 1984). Our perception and interpretation of touch are greatly influenced by context, expectations, and sensory characteristics (Sailer & Leknes, 2022). This explains why the experience and acceptance of touch vary within individuals (Bartz et al., 2011).

On the other hand, it is important to remember that differences may exist across individuals: While many do value and seek social touch, some individuals find it less pleasant or even unbearable. This may be due to cognitive differences (e.g., for autistic people; Gallace & Spence, 2010), pathological causes (e.g., people with anorexia nervosa; Crucianelli et al., 2016, 2021), or due to various personal and social factors, such as upbringing and culture. But even for such individuals, chosen touch may be less unpleasant than unchosen touch. Importantly, these observations tend to be made in contexts where the patients or participants have little or no control over the touch they receive (at least on a moment-to-moment basis, besides the general consent to be touched). Our study suggests that by providing people with a higher level of perceived control over where or how they are touched can significantly enhance their perceived pleasantness. Whether these effects would also extend to vulnerable populations or clinical settings is an important next step. Physical touch in medical settings has been shown to have a positive impact on people’s health (Henricson et al., 2008). By enhancing the positive experience of touch, the therapy outcome could be improved further.

Several limitations must be taken into account when interpreting this experimental study. Though too much choice could get tiring (Iyengar & Lepper, 2000), the sequential nature of the choices in our study, including alternating with no-choice trial blocks, did not seem to affect the extent to which people valued the chosen touch. Obviously, any practical implementation, say in therapeutic interventions, would need to revisit how frequent and repeated choice should be. As with many experiments in interpersonal touch, we focused on a young cohort. Ours predominantly consisted of males, and both of our experimenters were male, so age diversity, gender and interpersonal orientations could be important for future research to consider. Such factors could modulate when and how much choice affects which touch, but we do not expect them to make the effects of choice disappear. Controlling for gender in supplementary analyses did not alter our main conclusions. Existing research still warns that the choice-induced effects could be modulated by cultural background: Only German citizens participated in the present study, and it is possible that choice plays a stronger or weaker role in other cultures (Iyengar & Lepper, 1999). Additionally, we recognize the need for more research that examines the relationship between choice and perception of control as well as a wider range of physiological measures to gain a better understanding of the complex relationship between choice, touch experiences and psychological factors.

## Conclusion

The fact that choice positively influences the perception of touch, including when it concerns less relevant aspects of touch (though to a weaker extent), suggests that enhancing patients' sense of control could support better acceptance of therapies. It also extends the benefits of choice on reward beyond the classic free-choice paradigms which show an increased distance between a chosen and a rejected option. While such paradigms rest on the chooser being able to compare both external rewards before and after choice, our design did not rest on comparison and worked by offering choice only over a single intrinsic reward.

## Supplementary Information

Below is the link to the electronic supplementary material.

Supplementary file1 (PDF 109 KB)

## Appendix 1: Questionnaires

In the following appendix, we describe briefly the questionnaires we used and the appropriate analysis outcomes. To view the overall descriptive results of these questionnaires, please visit https://osf.io/jgknx.

### Social touch questionnaire

#### What attitudes did participants hold towards social touch?

An assessment of participants' attitudes toward touch in social situations was conducted using the Social Touch Questionnaire (STQ, Wilhelm et al., 2001). The overall attitude of the group stood at a positive middle ground in terms of a social touch (mean=42.304, SD=11.158). An STQ score of 80 indicates a relatively negative attitude towards the touch, while an STQ score of 20 indicates a relatively positive attitude towards the touch. Further analysis of the STQ items related to either giving and receiving touch (GIVE/GET) was conducted, eliminating two questions due to their ambiguity. No significant differences have been found between receiving and giving touch (meanGet=19.61, SD=6.415; meanGive=19.6, SD = 5.1, t(22) =-0.039, *p* = .969).

### Longing interpersonal touch picture questionnaire

#### What amount of touch did our participants experience on a daily basis?

As a way of assessing touch behaviour, participants were asked to describe their longing for a touch from their partners, family, or strangers. We administered the Longing Interpersonal Touch Picture Questionnaire (LITPQ, Beßler et al., 2019). Three touch types most relevant to our study ("forearm", "hugs", and "shoulders") were selected from the LITPQ. As a first step, touch frequency and touch wanting values were added separately across all types of touch per participant and then scores for wanting touch were divided by the score for frequency of getting touch. These scores were then averaged and the new CHOICE BOLSTERS TOUCH PLEASANTNESS 2 LITPQ coefficient number across all the participants was determined. A score below 1 indicates satisfaction with touch, while a score greater than 1 indicates a longing for touch.

According to the final LITPQ score for our group, a coefficient greater than 1 indicates an overall longing for touch (mean= 1.87, SD = 3.41).

### Spielberger state-trait anxiety inventory

#### How anxious were the participants before and after the experiment?

The Spielberger State-Trait Anxiety Inventory (STAI-6, Marteau & Bekker, 1992) STAI-6 Questionnaire was taken by the participants before and after the experiment to evaluate their level of anxiety. The group results indicated significantly higher levels of anxiety following the experiment (meanPost = 35.4, SD = 9.89) than before it (meanPre =30.9, SD = 7.73, t(22) = -2.345, *p*=0.029). A minimum score of 20 is indicative of a low anxiety level, whereas a maximum score of 80 indicates a high level.
