# Supplementary material for: Choice enhances touch pleasantness
Source: Atten Percept Psychophys. 2024 Jun 10;86(5):1709–23. doi: 10.3758/s13414-024-02887-6 (PMC11557629; doi:10.3758/s13414-024-02887-6)
Supplement: Supplementary file 1 — Supplementary file1 (PDF 109 KB) [file 13414_2024_2887_MOESM1_ESM.pdf]

Supplementary Material

The following tables summarise the model coefficients reported in the main text.

Table S1

Linear mixed-effects regression (LMER) coefficients for pleasantness ratings as a function of choice, relevance and speed (with questionnaire responses as covariates)

	Estimate	Std. Error	*t*-value	*p*-value	
(Intercept)	4.26	0.65	6.51	< 0.001	***
choice[yes]	0.08	0.09	0.95	0.349	
speed[suboptimal]	-1.64	0.29	-5.65	< 0.001	***
relevance[high]	-0.08	0.08	-1.03	0.310	
STQ	-0.00	0.01	-0.19	0.848	
LITPQ (quotient)	0.06	0.03	1.70	0.106	
STAI (pre)	0.01	0.01	0.76	0.458	
choice[yes]:speed[subopt.]	0.09	0.08	1.05	0.293	
choice[yes]:relevance[high]	0.22	0.08	2.67	0.008	**
speed[subopt.]:relevance[high]	0.22	0.08	2.67	0.008	**
choice[yes]:speed[subopt.]:rel.[high]	-0.17	0.12	-1.49	0.137	

Note: STQ: Social Touch Questionnaire; LITPQ: Longing for Interpersonal Touch Picture Questionnaire; STAI: Spielberger State-Trait Anxiety Inventory. See online analyses scripts (https://osf.io/jgknx/) for details, including random effects.

* $p < 0.05$, ** $p < 0.01$, *** $p < 0.001$.

Table S2

Cumulative mixed-effects model coefficients for pleasantness, modelled as an ordinal variable with a logit link, as a function of choice, relevance and speed (with questionnaire responses as covariates)

	Estimate	Std. Error	*z*-value	*p*-value	
Threshold coefficients					
Threshold[1|2]	-7.67	1.88	-4.08		
Threshold[2|3]	-5.09	1.88	-2.71		
Threshold[3|4]	-2.15	1.87	-1.15		
Threshold[4|5]	0.75	1.87	0.40		
Threshold[5|6]	3.64	1.87	1.94		
Threshold[6|7]	6.62	1.88	3.52		
Fixed effect coefficients					
choice[yes]	0.27	0.26	1.05	0.295	
speed[suboptimal]	-4.82	0.86	-5.59	< 0.001	***
relevance[high]	-0.14	0.22	-0.64	0.521	
STQ	-0.00	0.03	-0.07	0.943	
LITPQ (quotient)	0.15	0.11	1.42	0.155	
STAI (pre)	0.03	0.05	0.69	0.493	
choice[yes]:speed[subopt.]	0.21	0.24	0.87	0.385	
choice[yes]:relevance[high]	0.56	0.24	2.35	0.019	*
speed[subopt.]:relevance[high]	0.49	0.24	2.07	0.039	*
choice[yes]:speed[subopt.]:rel.[high]	-0.32	0.34	-0.96	0.338	

Note: STQ: Social Touch Questionnaire; LITPQ: Longing for Interpersonal Touch Picture Questionnaire; STAI: Spielberger State-Trait Anxiety Inventory. See online analyses scripts (https://osf.io/jgknx/) for details, including random effects, and for an equivalent model with a probit link function.

* p < 0.05, ** p < 0.01, *** p < 0.001.

Table S3

Linear mixed-effects regression (LMER) coefficients for pleasantness ratings as a function of choice, relevance, speed and participant gender (with questionnaire responses as covariates)

	Estimate	Std. Error	*t*-value	*p*-value	
Gender included as main effect only					
(Intercept)	4.28	0.72	5.97	< 0.001	***
choice[yes]	0.08	0.09	0.95	0.349	
speed[suboptimal]	-1.65	0.29	-5.65	< 0.001	***
relevance[high]	-0.08	0.08	-1.03	0.309	
STQ	-0.00	0.01	-0.20	0.843	
LITPQ (quotient)	0.06	0.04	1.63	0.121	
STAI (pre)	0.01	0.02	0.73	0.477	
gender[female]	-0.02	0.30	-0.07	0.942	
choice[yes]:speed[subopt.]	0.09	0.08	1.05	0.293	
choice[yes]:relevance[high]	0.22	0.08	2.67	0.008	**
speed[subopt.]:relevance[high]	0.22	0.08	2.67	0.008	**
choice[yes]:speed[subopt.]:rel.[high]	-0.17	0.12	-1.49	0.137	
Interactions between gender and experimental conditions					
(Intercept)	4.28	0.73	5.90	< 0.001	***
choice[yes]	0.13	0.10	1.29	0.205	
speed[suboptimal]	-1.66	0.34	-4.93	< 0.001	***
relevance[high]	-0.06	0.09	-0.60	0.553	
gender[female]	-0.01	0.54	-0.02	0.983	
STQ	-0.00	0.01	-0.20	0.843	
LITPQ (quotient)	0.06	0.04	1.63	0.121	
STAI (pre)	0.01	0.02	0.73	0.477	
choice[yes]:speed[subopt.]	0.10	0.09	1.09	0.276	
choice[yes]:relevance[high]	0.19	0.09	2.03	0.042	*
speed[subopt.]:relevance[high]	0.22	0.09	2.33	0.020	*
choice[yes]:gender[female]	-0.21	0.22	-0.98	0.330	
speed[subopt.]:gender[female]	0.06	0.72	0.08	0.937	
relevance[high]:gender[female]	-0.13	0.20	-0.64	0.525	
choice[yes]:speed[subopt.]:rel.[high]	-0.26	0.13	-1.96	0.050	*
choice[yes]:speed[subopt.]:gender[f.]	-0.07	0.20	-0.34	0.732	
choice[yes]:rel.[high]:gender[f.]	0.14	0.20	0.72	0.474	
speed[subopt.]:rel.[high]:gender[f.]	0.02	0.20	0.08	0.937	
choice[yes]:speed[subopt.]:rel.[high]:gender[f.]	0.39	0.28	1.39	0.166	

Note: STQ: Social Touch Questionnaire; LITPQ: Longing for Interpersonal Touch Picture Questionnaire; STAI: Spielberger State-Trait Anxiety Inventory. See online analyses scripts (https://osf.io/jgknx/) for details, including random effects.

* $p < 0.05$, ** $p < 0.01$, *** $p < 0.001$.

Table S4

Generalized additive mixed-effects model (GAMM) coefficients for z-scored pupil size during the anticipation phase, as a function of choice and relevance (with questionnaire responses as covariates)

	Estimate	Std. Error	*t*-value	*p*-value	
(Intercept)	0.13	0.56	0.23	0.819	
choice[yes]	0.56	0.09	6.31	< 0.001	***
relevance[high]	0.19	0.10	1.84	0.065	
STQ	-0.00	0.01	-0.41	0.680	
STAI (pre)	-0.00	0.01	-0.21	0.837	
LITPQ (get)	-0.01	0.02	-0.56	0.574	
LITPQ (want)	-0.00	0.01	-0.21	0.835	
choice[yes]:relevance[high]	-0.13	0.00	-43.63	< 0.001	***
LITPQ (get):LITPQ (want)	0.00	0.00	0.31	0.757	

Note: STQ: Social Touch Questionnaire; LITPQ: Longing for Interpersonal Touch Picture Questionnaire; STAI: Spielberger State-Trait Anxiety Inventory. See online analyses scripts (https://osf.io/jgknx/) for details, including random effects and model smooth terms.

* p < 0.05, ** p < 0.01, *** p < 0.001.

Table S5

Generalized additive mixed-effects model (GAMM) coefficients for z-scored and baseline-corrected pupil size during the anticipation phase, as a function of choice and relevance (with questionnaire responses as covariates)

	Estimate	Std. Error	*t*-value	*p*-value	
(Intercept)	-0.01	0.31	-0.03	0.978	
choiceyes	0.55	0.06	8.91	< 0.001	***
relevancehigh	-0.05	0.04	-1.20	0.230	
STQ	-0.00	0.00	-0.20	0.843	
STAI (pre)	-0.01	0.01	-1.60	0.111	
LITPQ (get)	0.00	0.01	0.28	0.781	
LITPQ (want)	0.00	0.01	0.12	0.903	
choice[yes]:relevance[high]	-0.01	0.00	-2.49	0.013	**
LITPQ (get):LITPQ (want)	-0.00	0.00	-0.54	0.589	

Note: STQ: Social Touch Questionnaire; LITPQ: Longing for Interpersonal Touch Picture Questionnaire; STAI: Spielberger State-Trait Anxiety Inventory. See online analyses scripts (https://osf.io/jgknx/) for details, including random effects and model smooth terms.

* $p < 0.05$, ** $p < 0.01$, *** $p < 0.001$.

Table S6

Generalized additive mixed-effects model (GAMM) coefficients for z-scored pupil size during the touch phase, as a function of choice, relevance and speed (with questionnaire responses as covariates)

	Estimate	Std. Error	t-value	p-value	
(Intercept)	0.25	0.48	0.51	0.612	
choice[yes]	0.31	0.08	4.15	< 0.001	***
relevance[high]	0.26	0.09	2.76	0.006	**
speed[optimal]	-0.10	0.04	-2.64	0.008	**
STQ	0.00	0.01	0.10	0.917	
STAI (pred)	0.00	0.01	-0.00	0.999	
LITPQ (get)	0.00	0.01	0.14	0.889	
LITPQ (want)	-0.00	0.01	-0.25	0.804	
choice[yes]:relevance[high]	-0.18	0.00	-72.65	< 0.001	***
choice[yes]:speed[opt.]	-0.00	0.00	-1.41	0.159	
relevance[high]:speed[opt.]	0.05	0.00	21.15	< 0.001	***
choice[yes]:rel.[high]:speed[opt.]	-0.03	0.00	-8.08	< 0.001	***

Note: STQ: Social Touch Questionnaire; LITPQ: Longing for Interpersonal Touch Picture Questionnaire; STAI: Spielberger State-Trait Anxiety Inventory. See online analyses scripts (https://osf.io/jgknx/) for details, including random effects and model smooth terms.

* $p < 0.05$, ** $p < 0.01$, *** $p < 0.001$.

Table S7

Generalized additive mixed-effects model (GAMM) coefficients for z-scored and baseline-corrected pupil size during the touch phase, as a function of choice, relevance and speed (with questionnaire responses as covariates)

	Estimate	Std. Error	t-value	p-value	
(Intercept)	0.55	0.40	1.37	0.171	
choice[yes]	-0.60	0.08	-7.32	< 0.001	***
relevance[high]	-0.08	0.05	-1.47	0.141	
speed[optimal]	-0.11	0.04	-2.85	0.004	**
STQ	0.00	0.01	0.39	0.695	
STAI (pre)	0.00	0.01	0.32	0.746	
LITPQ (get)	0.01	0.01	1.11	0.266	
LITPQ (want)	-0.00	0.01	-0.72	0.472	
choice[yes]:relevance[high]	0.00	0.00	0.31	0.758	
choice[yes]:speed[opt.]	0.03	0.00	13.32	< 0.001	***
relevance[high]:speed[opt.]	0.17	0.00	72.49	< 0.001	***
choice[yes]:rel.[high]:speed[opt.]	-0.14	0.00	-44.63	< 0.001	***

Note: STQ: Social Touch Questionnaire; LITPQ: Longing for Interpersonal Touch Picture Questionnaire; STAI: Spielberger State-Trait Anxiety Inventory. See online analyses scripts (https://osf.io/jgknx/) for details, including random effects and model smooth terms.

* p < 0.05, ** p < 0.01, *** p < 0.001.

Table S8

Linear mixed-effects regression (LMER) coefficients for first peak in z-scored pupil size identifiable after t = 220ms in the touch phase, as a function of choice, relevance and speed (with questionnaire responses as covariates)

	Estimate	Std. Error	*t*-value	*p*-value	
(Intercept)	0.36	0.30	1.19	0.245	
relevance[low]	-0.11	0.14	-0.75	0.460	
choice[yes]	0.40	0.10	3.92	< 0.001	***
speed[suboptimal]	0.06	0.07	0.91	0.363	
STQ	-0.00	0.00	-0.22	0.829	
STAI (pre)	-0.00	0.01	-0.41	0.684	
LITPQ (get)	-0.00	0.01	-0.69	0.502	
LITPQ (want)	-0.00	0.00	-0.09	0.931	
relevance[low]:choice[yes]	0.03	0.10	0.34	0.733	
relevance[low]:speed[subopt.]	-0.06	0.10	-0.60	0.547	
choice[yes]:speed[subopt.]	0.05	0.10	0.49	0.626	
rel.[low]:choice[yes]:speed[subopt.]	-0.01	0.14	-0.11	0.916	

Note: STQ: Social Touch Questionnaire; LITPQ: Longing for Interpersonal Touch Picture Questionnaire; STAI: Spielberger State-Trait Anxiety Inventory. See online analyses scripts (https://osf.io/jgknx/) for details, including random effects.

* $p < 0.05$, ** $p < 0.01$, *** $p < 0.001$.

Table S9

Linear mixed-effects regression (LMER) coefficients for first peak in z-scored and baseline-corrected pupil size identifiable after t = 220ms in the touch phase as a function of choice, relevance and speed (with questionnaire responses as covariates)

	Estimate	Std. Error	*t*-value	*p*-value	
(Intercept)	0.54	0.29	1.88	0.075	
relevance[low]	0.06	0.07	0.89	0.376	
choice[yes]	-0.46	0.08	-5.48	< 0.001	***
speed[suboptimal]	-0.08	0.06	-1.38	0.166	
STQ	0.00	0.00	1.20	0.245	
STAI (pre)	-0.00	0.01	-0.59	0.561	
LITPQ (get)	0.01	0.01	1.10	0.287	
LITPQ (want)	-0.00	0.00	-1.03	0.317	
relevance[low]:choice[yes]	0.01	0.08	0.15	0.881	
relevance[low]:speed[subopt.]	0.13	0.08	1.55	0.121	
choice[yes]:speed[subopt.]	0.17	0.08	2.15	0.032	*
rel.[low]:choice[yes]:speed[subopt.]	-0.19	0.11	-1.64	0.100	

Note: STQ: Social Touch Questionnaire; LITPQ: Longing for Interpersonal Touch Picture Questionnaire; STAI: Spielberger State-Trait Anxiety Inventory. See online analyses scripts (https://osf.io/jgknx/) for details, including random effects.

* $p < 0.05$, ** $p < 0.01$, *** $p < 0.001$.
